# Supplementary material for: Acoustically actuated ultra-compact NEMS magnetoelectric antennas
Source: Nat Commun. 2017 Aug 22;8:296. doi: 10.1038/s41467-017-00343-8 (PMC5567369; doi:10.1038/s41467-017-00343-8)
Supplement: Supplementary file 1 — Supplementary Information [file 41467_2017_343_MOESM1_ESM.pdf]

Description of Supplementary Files

File Name: Supplementary Information
Description: Supplementary Figures and Supplementary Notes.

File Name: Peer Review File

Supplementary Note 1. FeGaB/Al_2O_3 magnetic multilayer characterizations:

The magnetic properties of AlN/FeGaB/Al_2O_3 multilayers have been characterized both statically and dynamically by a vibration sample magnetometer (VSM) and ferromagnetic resonance spectroscopy (FMR) as shown in Supplementary Fig. 1. The sample has the same stacking as reported in the main text (500 nm AlN and [$Fe_7Ga_2B_1$ (45 nm)/Al_2O_3 (5 nm)] ×10). Instead of the patterned devices, the reference sample is a full film with a lateral dimension of 5 mm by 5 mm. Note that there could be a variation in magnetic properties between the reference sample and device due to the different shape anisotropy and stress state. As shown in Supplementary Fig. 1(a) the magnetic hysteresis of FeGaB/Al_2O_3 multilayers with a magnetic coercive field <0.5 mT indicating a soft magnetic property. This is important for achieving large magnetostriction constant and self-bias property of the proposed devices. Supplementary Fig. 1(b) shows the FMR spectrum taken at 9.5 GHz of FeGaB/Al_2O_3 multilayers which gives a resonance frequency of 93 mT and magnetic moment of 1.15 T based on the Kittel equation. The resonance linewidth of 6 mT can also be obtained demonstrating a good microwave property with a low magnetic loss.

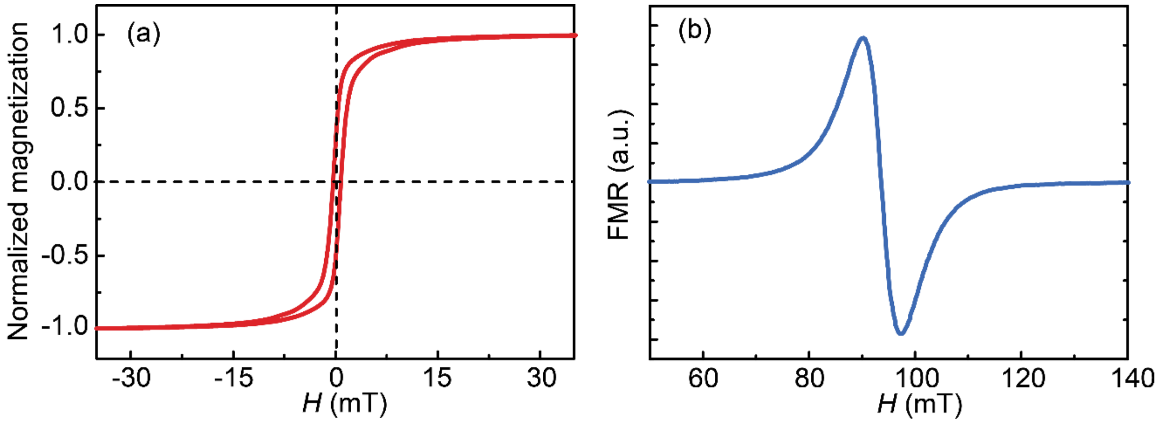

Supplementary Figure 1. Magnetic properties of FeGaB/Al_2O_3 multilayers. Magnetic hysteresis loop (a) and Ferromagnetic resonance spectrum (b) of FeGaB/Al_2O_3 multilayers.

Supplementary Note 2. Device fabrication process and FBAR device layout:

The nano-plate resonator (NPR) and thin-film bulk acoustic wave resonator (FBAR) devices share the same fabrication process as shown in Supplementary Fig. 2. The process starts with a high resistivity Silicon (Si) wafer (>10000 ohm cm). A 50 nm thick Pt film was sputter-deposited and patterned by lift-off on top of the Si substrate to define the bottom electrodes (a). Then, the 500 nm AlN film was sputter-deposited and vias to access the bottom electrodes etched by H_3PO_4 (b). After that, the AlN film was etched by Inductively Coupled Plasma (ICP) etching in Cl_2 based chemistry to define the shape of the resonant nano-plate (c). Next, a 100 nm thick gold (Au) film was evaporated and patterned by lift-off to form the top ground (d). Finally, 500 nm thick FeGaB/Al_2O_3 multilayer layer was deposited by a magnetron sputtering and patterned by lift-off (e). A 100 Oe in-situ magnetic field bias was applied during the magnetic deposition along the width direction of the device to pre-orient the magnetic domains. Then, the structure was released by XeF_2 isotropic etching of the Silicon substrate (e). Supplementary Fig. 2f shows the device layout of the magnetoelectric (ME) FBAR antenna with the detailed dimensions.

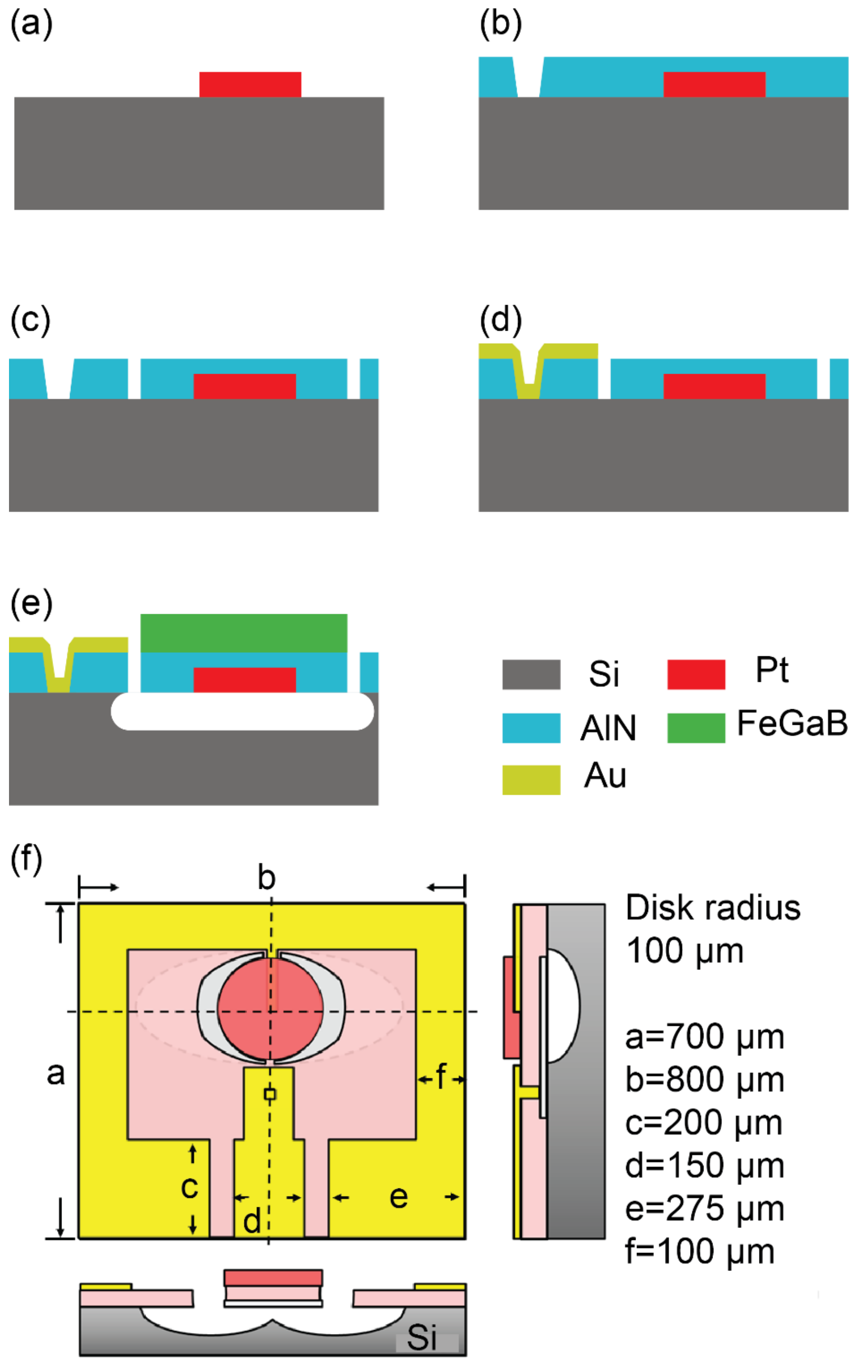

Supplementary Figure 2. NPR and FBAR devices fabrication process. (a) Pt thin film deposition and patterning. (b) AlN thin film deposition and wet etch of vias. (c) AlN thin film ICP etching. (d) Top electrode Au deposition and patterning. (e) Magnetic multilayer FeGaB/Al_2O_3 deposition and pattering followed by Si substrate release. (f) Device layout of the ME FBAR antenna.

Supplementary Note 3. Equivalent circuit modeling of NPR antenna:

As shown in Fig. 1 (c), the admittance amplitude of NPR can be fitted with Butterworth–van Dyke (BVD) model to extract the electromechanical parameters such as electromechanical coupling coefficient k_t^2 and Q-factor. BVD equivalent circuit consists of electrical components and equivalent mechanical components connected in parallel. As shown in Supplementary Fig. 3, the electrical components include the device capacitance C_0 which is defined by the device geometry and a resistance R_{0p} which is associate with the dielectric loss. While the mechanical branch contains the motional capacitance C_m, motional inductance L_m and motional resistance R_m, which can be expressed as $R_m = \frac{1}{\omega_0 C_0 k_t^2 Q}$, $C_m = \frac{8}{\pi^2} C_0 k_t^2$, and $L_m = \frac{1}{{\omega_0}^2 C_m}$. The series resistance R_s is serial connected to both branches as the electrical loss of the electrodes. The resonance frequency occurs at the resonance frequency $2\pi\omega_0$, where the C_m and L_m cancel with each other. The kt^2 represents the efficiency of electrical and acoustic energy conversion, and Q-factor defines the ratio of the energy stored in the vibrating resonant structure to the energy dissipated per cycle by the damping processes. Note that the $k_t^2 \cdot Q$ is the figure of merit (FOM) of an electromechanical resonator.

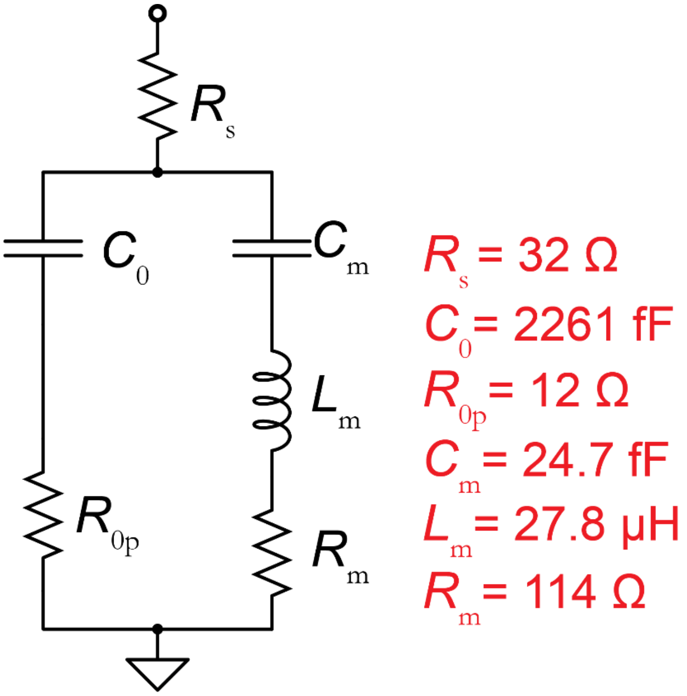

Supplementary Figure 3. Equivalent BVD model circuit and the fitting parameters of ME NPR device shown in Fig.1 (c).

Supplementary Note 4. Simulation of RF magnetic field:

The RF magnetic field is this work is generated by a RF coil with an inner diameter of 7.7 mm and 7 turns. The RF coil was soldered on SMA port and connected to the out-put port of the lock-in amplifier. The RF magnetic field (H_{rf}) generated by the RF coil is simulated by Finite Element Method (FEM) software, Comsol Multiphysics V5.1. The input is RF voltage with a peak-peak amplitude of 1 V. Supplementary Fig. 4a shows the $\mu_0 H_{rf}$ as a function of the center axis coordinate (x). The left end of the coil is placed at x=0 mm, and the NPR devices under test (DUT) is placed at x= -14 mm. At this scenario, the $\mu_0 H_{rf}$ applied to the DUT is 60 nT. Supplementary Fig. 4b shows the $\mu_0 H_{rf}$ as a function of frequency. A small frequency dependency was observed due to the impedance change of the RF coil.

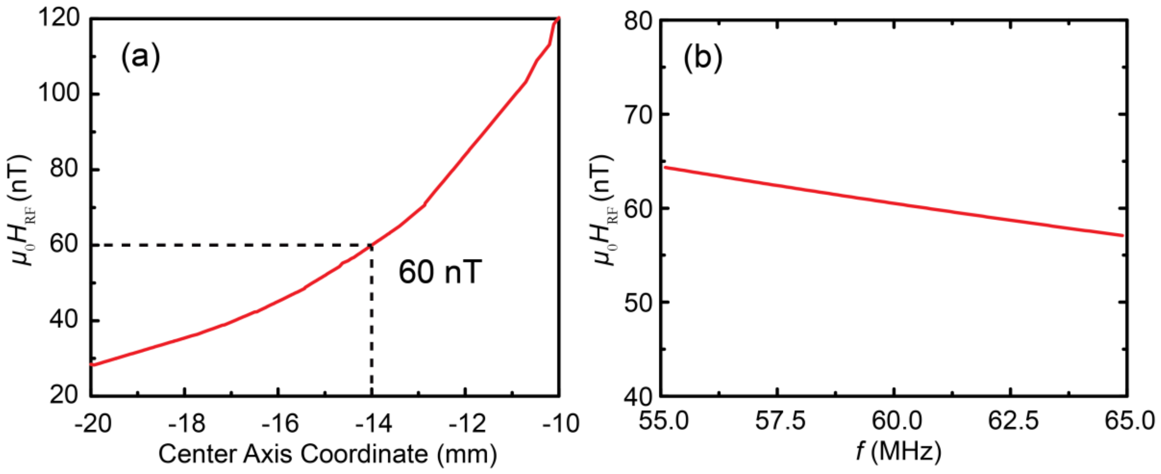

Supplementary Figure 4. (a) RF magnetic field ($\mu_0 H_{rf}$) versus center axis coordinate (x). The device under test (DUT) is placed at x=-14 mm with respect to the RF coil, which gives a $\mu_0 H_{rf}$ of 60 nT at 60.7 MHz. (b) $\mu_0 H_{rf}$ as a function of frequency.

Supplementary Note 5. Induced ME voltage on the multi-finger interdigital NPR:

We also tested the interdigital multi-finger NPR with the same AlN/FeGaB ME heterostructure. There are 3 interdigital electrodes with each digital electrode having a width of 30 μm and a length of 200 μm. By the admittance amplitude measurements, the resonance frequency and quality factor was found to be 139 MHz and 500. The ME voltage was measured with the same experimental setup as the single finger NPR device (as shown in fig. 1f), however no noticeable signal can be picked up around 139 MHz. As we discussed in the main manuscript, the negligibly ME voltage can be attributed to the non-uniform induced strain. This can be verified by the FEA as shown in the Supplementary Fig. 5. The induced voltage at the resonance frequency is at least 3 orders of magnitudes lower than the single finger ME NPR device. The Supplementary Fig. 5 shows the in-plane

displacement excited by the RF magnetic field, which confirms the non-uniformity of the in-plane strain.

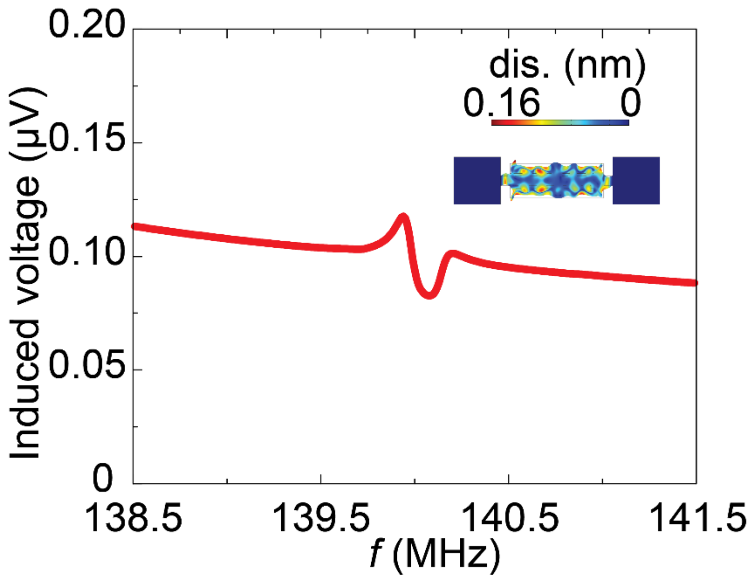

Supplementary Figure 5. FEA of ME multi-finger NPR for the induced ME voltage measurement. The inset shows the in-plane displacement excited by the RF magnetic field.

Supplementary Note 6. Single-chip, multi-band NPR and FBAR ME resonators:

We measured the resonance frequency f_r and Q-factor of various devices with different design principles and geometries via a network analyzer. Supplementary Fig. 6 plots the f_r and Q as the function of $1/W$ for NPR and $1/t$ for FBAR. All devices are fabricated on one single chip with the same fabrication and deposition processes, which have the same layered structure (500 nm AlN and [$Fe_7Ga_2B_1$ (45 nm)/Al_2O_3 (5 nm)] ×10) as the devices discussed in the main text. As discussed in the main text, the f_r of NPR devices is inversely

proportional to the *W* for NPRs, which cover a wide range of frequency bands continuously from 60 MHz to 1500 MHz. For FBAR, its f_r is inversely proportional to the AlN thickness, exhibiting a f_r of 2500 MHz. In addition, the *Q*-factors are high for all devices. By simulation and device geometry design, we can achieve a very wide frequency band from tens of MHz (NPR with large *W*) to tens of GHz (FBAR with thinner AlN thickness) on one chip. A bank of multi-frequency MEMS resonators can be connected to a CMOS oscillator circuit for the realization of reconfigurable ME antenna array.

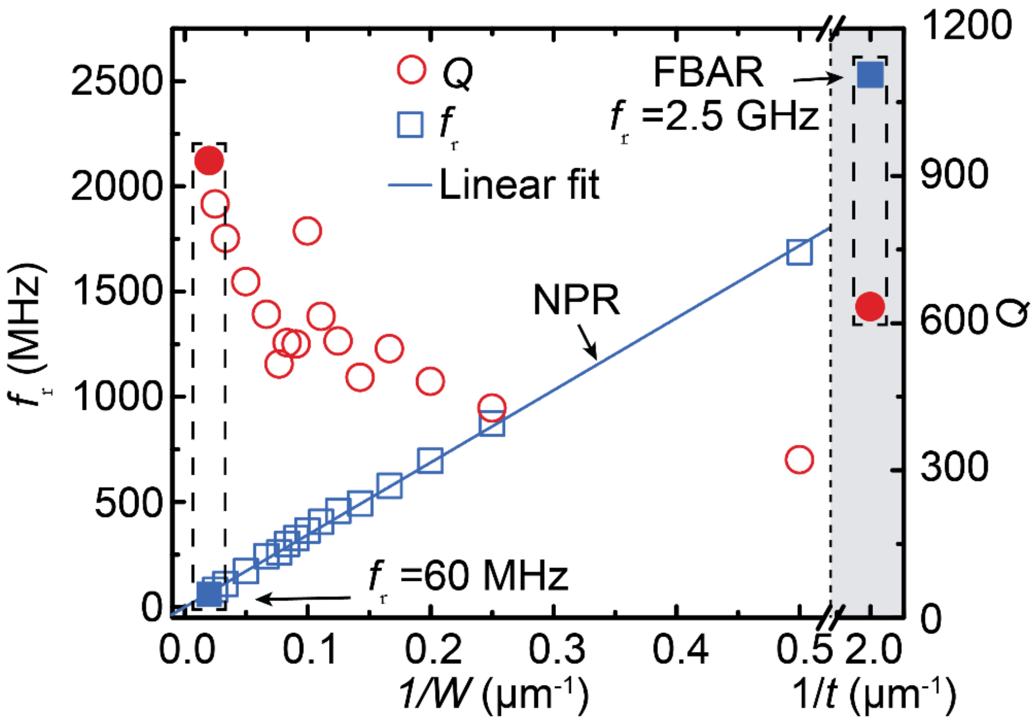

Supplementary Figure 6. Resonance frequency f_r and *Q*-factor as the function of one over device width (1/*W)* for NPR devices and one over AlN thickness for FBAR devices. The solid dots represent the data of the 100 μm width NPR and the 500 nm thick FBAR devices in the main paper.

Supplementary Note 7. Simulation of the electromechanical property of FBAR device:

Piezoelectric module in Comsol Multiphysics was used for the electromechanical simulation of the FBAR device. Supplementary Figure 7 shows the simulated reflection coefficient (S_{22}) of the FBAR device showing the estimated electromechanical resonance frequency of 2.52 GHz.

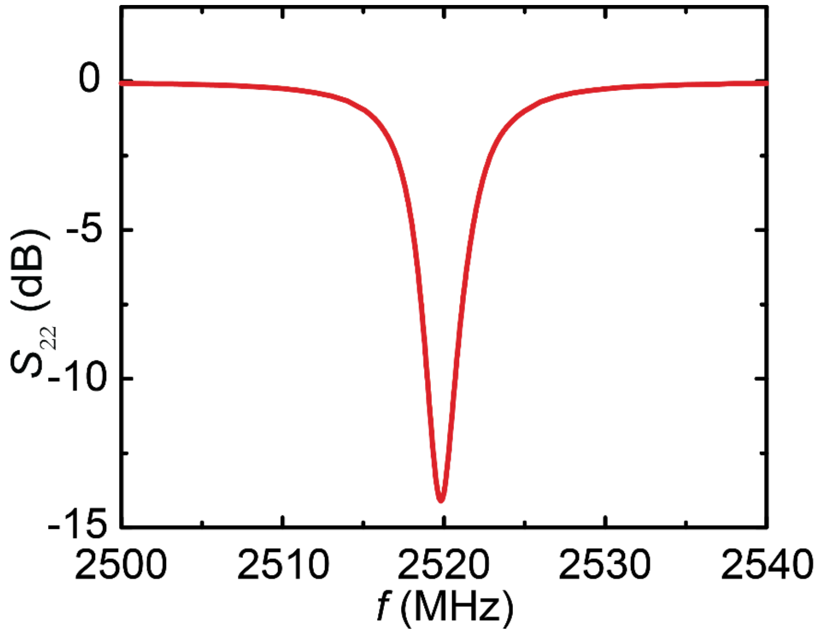

Supplementary Figure 7. Simulated reflection coefficient (S_{22}) of the FBAR device.

Supplementary Note 8. Simulation of the small loop antenna:

Small loop antennas have overall circumference less than about one-tenth of a wavelength (C< λ_0/10). They have small radiation resistance which are usually smaller than their loss resistances. Low radiation resistance and high reactance make their impedance matching

difficult. Small loop antennas are most often used as receive antennas for magnetic field sensing or magnetic radiators, where impedance mismatch loss can be tolerated. ANSYS HFSS 15 is used to simulate the performance of the small loop antenna. The small loop antenna has the same dimension of the ME FBAR antenna including the ground loop with a=550 μm, where a is the smallest imaginary sphere of radius enclosed the entire antenna structure. The small loop antenna was design as chip-scale devices and compatible with a lithographic fabrication process. The substrate is a 2.2 μm thick AlN and the conductor is a 5 μm thick copper. The reflection peak at 34 GHz has a return loss of -22 dB and Q-factor <10. At 2.52 GHz (the resonance frequency of ME FBAR antenna), the return loss is about -0.065 dB.

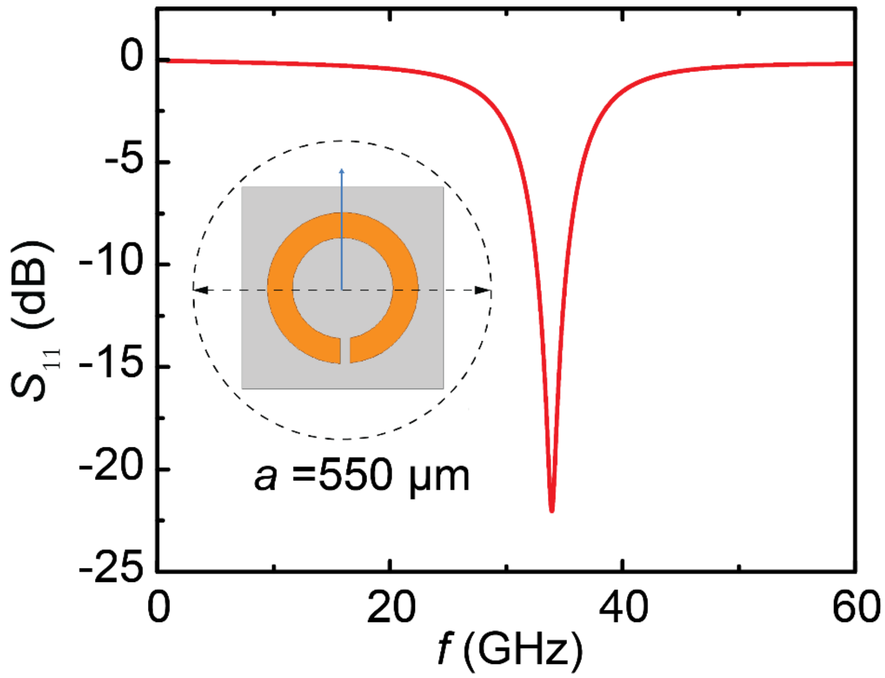

Supplementary Figure 8. Simulated reflection coefficient (S_{11}) of the small loop antenna. The inset shows the schematic of the simulated small loop antenna.

Supplementary Note 9. Magnetoelectric Coupling and Frequency Analysis:

To analyze the response of the ME structures, the coupling between the magnetic, elastic and electric field in the two different magnetostrictive and piezoelectric should be taken into consideration. Simulations with FEM software, Comsol Multiphysics V5.1 were carried out to investigate the frequency response analysis by existing modules which are magnetic fields, solid mechanics, and electrostatics modules. The ME composites were constructed into magnetostrictive, piezoelectric phase and air subdomain and performed with the frequency domain in 3D geometry in order to illustrate the modeling principles for more complicated problems.

In air phase, we assumed that a spatially uniform, sinusoidal wave magnetic field is applied. The air model space is truncated by an infinite element domain region. When using the infinite element domain features, the boundary conditions on the outside of the modeling does not affect the solutions.

In the magnetostrictive FeGaB, the magnetic permeability and the magnetostrictive strains, shows nonlinear dependency on the magnetic flux and the mechanical stress/strains in the ME composite. The constitutive equations of the magnetostrictive has been performed with an experimental measured nonlinear magentostriction versus magnetic field. The magnetic flux density (***B)*** versus H relationship can be separated as two part, one in the non-magnetic phase, such as piezoelectric and air, with $\boldsymbol{B} = \mu_0 \cdot \mu_{\mathrm{r}} \cdot H$. For magnetostatic approximation, we simply used a built in Maxwell equations in magnetic field modules:

$$\nabla \times H = j\omega D + J \qquad (1)$$

$$\nabla \times E = -j\omega B - M \qquad (2)$$

Where D is the electric flux density, J and M are the electric current density and magnetic current density.

Another one is in the magnetostrictive phase with $B=f(H)$ with an interpolation function based on experimental results.

For the piezoelectric AlN, we assume a small signal behavior descried by the linear piezo material model, in which we established constitutive relations in a strain-charge form. Similarly, piezoelectric tensors and mechanical properties were obtained from the built-in condition for the PZD modules. The relation between the stress, electric field and electric displacement field in a stress-charge form is given by the piezoelectric constitutive equations,

$$\sigma = c\varepsilon - eE \qquad (3)$$

$$D = c\varepsilon + \kappa E \qquad (4)$$

where σ and ε are the stress and strain tensors, E and D are the electric filed and electric flux density. c, e and κ are the stiffness, strain to electric field coupling constant and permittivity, respectively. The solid mechanics model is described by the elastic constitutive relations,

$$\varepsilon = \frac{1}{2}\left[\left(\nabla u\right)^T + \nabla u\right] \qquad (5)$$

$$\sigma = C\varepsilon \qquad (6)$$

and

$$\nabla\sigma = -\rho\omega^2 \qquad (7)$$

where u is the displacement, ρ is the density, ω is the angular frequency, and C is the elasticity matrix.

The electrostatics model for modeling a piezoelectric in Comsol Multiphysics provides the following equations,

$$\nabla \cdot \mathrm{D} = \rho_v \quad (8)$$

$$E = -\nabla \varphi \quad (9)$$

where ρ_v is the electric charge density, and φ is the electric potential.

In the simulation, we used the parameters of the AlN from Comsol Multiphysics material library, which is listed at Supplementary Note 10. For simplification, we modeled the magnetostrictive by using a linear elastic model which is a part of PZD modules.

Supplementary Note 10. The linear materials parameters:

AlN:

Relative Permeability is 1

Density: 3300 kg cm^{-3}

Electrical Conductivity: 10^{-6} S m^{-1}

Dielectric loss factor is 0.002

Mechanical damping loss factor is 0.001

Elasticity in matrix:

Supplementary Information

$$\begin{pmatrix} 41 & 14.9 & 9.9 & 0 & 0 & 0 \\ 14.9 & 41 & 9.9 & 0 & 0 & 0 \\ 9.9 & 99 & 38.9 & 0 & 0 & 0 \\ 0 & 0 & 0 & 12.5 & 0 & 0 \\ 0 & 0 & 0 & 0 & 12.5 & 0 \\ 0 & 0 & 0 & 0 & 0 & 12.5 \end{pmatrix} 10^{10}\,\mathrm{Pa}\ .$$

Piezoelectric charge coupling constants in matrix:

$$\begin{pmatrix} 0 & 0 & 0 & 0 & -0.48 & 0 \\ 0 & 0 & 0 & -4.8 & 0 & 0 \\ -0.58 & -0.58 & 1.55 & 0 & 0 & 0 \end{pmatrix} Cm^2.$$

Relative permittivity in matrix:

$$\begin{pmatrix} 9.2 & 0 & 0 \\ 0 & 9.2 & 0 \\ 0 & 0 & 10.3 \end{pmatrix}.$$

FeGaB:

Density is 7860 kg cm^{-3}

Poisson’s ratio is 0.27.

Relative permittivity is 1.

Relative permeability is 1300.

Electrical Conductivity is 200000 S m^{-1}.

Young’s Modulus is 55 GPa.

Magnetostriction is 70 ppm.

Saturation magnetization is 1114084 A m^{-1}.
